# Spinal Cord Stimulation Attenuates Mechanical Allodynia and Increases Central Resolvin D1 Levels in Rats With Spared Nerve Injury

**DOI:** 10.3389/fphys.2021.687046

**Published:** 2021-06-25

**Authors:** Xueshu Tao, Xin Luo, Tianhe Zhang, Brad Hershey, Rosana Esteller, Ru-Rong Ji

**Affiliations:** ^1^Center for Translational Pain Medicine, Department of Anesthesiology, Duke University Medical Center, Durham, NC, United States; ^2^Department of Pain Medicine, The First Affiliated Hospital of China Medical University, Shenyang, China; ^3^Boston Scientific Neuromodulation Research and Advanced Concepts, Valencia, CA, United States; ^4^Department of Cell Biology, Duke University Medical Center, Durham, NC, United States; ^5^Department of Neurobiology, Duke University Medical Center, Durham, NC, United States

**Keywords:** spinal cord stimulation, specialized pro-resolving mediators, docosahexaenoic acid, resolving D1 (RvD1), Interleukin – 1 β, neuroinflammation, neuropathic pain, nerve injury

## Abstract

Mounting evidence from animal models of inflammatory and neuropathic pain suggests that inflammation regulates the resolution of pain by producing specialized pro-resolving mediators (SPMs), such as resolvin D1 (RvD1). However, it remains unclear how SPMs are induced in the central nervous system and whether these mechanisms can be reconciled with outcomes of neuromodulation therapies for pain, such as spinal cord stimulation. Here, we show that in a male rat model of neuropathic pain produced by spared nerve injury (SNI), 1 kHz spinal cord stimulation (1 kHz SCS) alone was sufficient to reduce mechanical allodynia and increase RvD1 in the cerebrospinal fluid (CSF). SNI resulted in robust and persistent mechanical allodynia and cold allodynia. Spinal cord electrode implantation was conducted at the T11-T13 vertebral level 1 week after SNI. The spinal locations of the implanted electrodes were validated by X-Ray radiography. 1 kHz SCS was applied for 6 h at 0.1 ms pulse-width, and this stimulation alone was sufficient to effectively reduce nerve injury-induced mechanical allodynia during stimulation without affecting SNI-induced cold allodynia. SCS alone significantly reduced interleukin-1β levels in both serum and CSF samples. Strikingly, SCS significantly increased RvD1 levels in the CSF but not serum. Finally, intrathecal injection of RvD1 (100 and 500 ng, i.t.) 4 weeks after nerve injury reduced SNI-induced mechanical allodynia in a dose-dependent manner. Our findings suggest that 1 kHz SCS may alleviate neuropathic pain via reduction of IL-1β and via production and/or release of RvD1 to control SNI-induced neuroinflammation.

## Introduction

Pain is typically associated with inflammation as one of five main cardinal symptoms of inflammation: rubor (redness), tumor (swelling), calor (increased heat), dolor (pain), and functio laesa (loss of function) (Tao et al., 2020). It is well appreciated that inflammation produces inflammatory mediators, such as prostaglandins, pro-inflammatory cytokines/chemokines to elicit pain (Sommer and Kress, 2004; Verri et al., 2006; Ji et al., 2014). Inflammatory mediators cause nociceptor sensitization (peripheral sensitization) by interacting with their receptors expressed by nociceptors. Accumulating evidence suggests that inflammation also plays an active role in the resolution of pain (Ji et al., 2011; Matsuda et al., 2019). A major advance in inflammation research is the appreciation that resolution of acute inflammation is not a passive process but an active biochemical programing that represents a new therapeutic frontier (Gilroy et al., 1999; Serhan, 2014; Fullerton and Gilroy, 2016). A milestone in “resolution biology” is the discovery of the specialized pro-resolving mediators (SPMs), which are generated during the resolution phase of inflammation and contribute importantly to the resolution process (Bannenberg and Serhan, 2010; Buckley et al., 2014). SPMs, such as resolvins, protectins, and maresins, are biosynthesized from omega-3 unsaturated fatty acids docosahexaenoic acid (DHA) and eicosapentaenoic acids (EPA) that are enriched in fish oil dietary supplements (Serhan, 2014). Increasing evidence has demonstrated potent analgesic actions of resolvins, such as resolvin D1 (RvD1) and E1 (RvE1) in animal models of inflammatory pain and neuropathic pain (Xu et al., 2010; Lima-Garcia et al., 2011; Fattori et al., 2020; Tao et al., 2020). However, it is still unclear how the production and release of SPMs are induced in the central nervous system.

Therapies for pain include but are not limited to pharmacological therapies and neuromodulation. Pharmacological therapies for pain are abundant and can target specific molecular mechanisms, pharmacologics non-topical pain are generally spatially non-specific in nature, and when given stand-alone, are often ineffective in treating refractory pain, with approximately 50% of patients still experiencing pain after a course of therapy (Finnerup et al., 2010). In contrast, neuromodulation involves the application of electricity directly to the neural substrate(s) responsible for pain control, with the intent of modulating neuronal and/or glial effects via spatially selective evoked activity and resultant downstream wholesale synaptic release rather than via the delivery of chemical agents. Spinal cord stimulation (SCS) has emerged as a viable form of neuromodulation for neuropathic pain in patients that has been practiced for over 50 years, demonstrating response (≥50% reduction in pain) in ∼50% of patients (Caylor et al., 2019). Traditionally clinicians have chosen to use lower stimulation pulse frequencies (30–60 Hz) but more recently, kilohertz (kHz) frequency sup-perception SCS has shown efficacy in patients refractory to conventional medical management (Krames et al., 2018) as well as in patients who experienced suboptimal relief using lower rate subperception SCS (North et al., 2016). Specifically, kHz sub-perception SCS resulted in improvement in more than 95% of patients compared to 41% who reported improvement following low frequency supra-perception SCS (North et al., 2016). Studies have shown that, in some cases, sub-perception SCS can reduce pain to the point that it could be considered in remission, raising possibility that the effects of SCS may be treating the underlying disease and promoting, at least temporarily, resolution of pain symptoms. While kHz frequencies are not always required to achieve profound sub-perception pain relief – a novel fast-acting sub-perception SCS therapy was shown to product nearly 80% reduction in pain intensity at 6-months (Metzger et al., 2021) – studies have shown that kHz frequency SCS improvements can be equally efficacious over a wide range of kHz frequencies, with 1 kHz SCS being the most energy-efficient in the 1–10 kHz range in a small double-blinded RCT (Thomson et al., 2018). However, the lack of paresthesia and slower analgesia onset time (Al-Kaisy et al., 2015) associated with kHz SCS suggest that mechanisms beyond traditional hypotheses regarding dorsal column activation, such as the Gate Control Theory, may contribute to its therapeutic effects (Crosby et al., 2017; Linderoth and Foreman, 2017).

Recent advances in pain research have revealed a critical role of spinal glial cells (e.g., microglia and astrocytes) and neuroinflammation in the pathogenesis of chronic pain, especially neuropathic pain (Peng et al., 2016; Chen et al., 2018; Inoue and Tsuda, 2018; Ji et al., 2019). Upon activation, spinal glial cells can produce pro-inflammatory cytokines such as IL-1β to elicit central sensitization and enhance pain states, glial cells can also produce anti-inflammatory cytokines and SPMs to promote the resolution of pain (Chen et al., 2018), but the roles of glia in the mechanism of action of SCS remain unclear. A possible modulation of spinal glial cell activity by low rate SCS (e.g., 50 or 60 Hz) has been implicated (Sato et al., 2014; Shu et al., 2020; Vallejo et al., 2020). Another report suggests that a special “multiplexed” waveform containing the specific combination of 50 Hz and 1,200 Hz SCS is necessary to elicit both behavioral improvements and anti-inflammatory effects by SCS following spared nerve injury (Vallejo et al., 2020), in contrast to others who have reported behavioral analgesia with either low rate or high rate SCS alone (Shechter et al., 2013; Song et al., 2014). The results with the “multiplexed” approach have caused significant uncertainty as to what SCS parameters are necessary or sufficient to generate both anti-inflammatory and analgesic effects. In this study, we used a rat model of neuropathic pain to investigate whether 1 kHz SCS alone could control neuropathic pain symptom via enhancing SPM production and simultaneously reducing IL-1β production and release in the CNS.

## Materials and Methods

### Animals

Sprague–Dawley rats, weighing 250–300 g, were purchased from Charles River Laboratories. Only male rats were used for behavioral and biochemical studies. Rats were group-housed on a 12-h light/12-h dark cycle at 22 ± 1°C with free access to food and water. Animals were randomly assigned to each group. Two rats were housed in each cage. All animal experiments were conducted in accordance with the National Institutes of Health Guide for the Care and Use of Laboratory Animals and approved by the Institutional Animal Care Use Committee of Duke University.

### Spared Nerve Injury Model of Neuropathic Pain

Neuropathic pain was induced by spared nerve injury (SNI) in rats. To produce SNI, the tibial and common peroneal nerves were tightly ligated with 5–0 silk, followed by transection and removal of 3–5 mm nerve segments, as previously described (Decosterd and Woolf, 2000; Wen et al., 2007). Caution was taken not to touch the sural nerve during surgery. In this model, neuropathic pain was measured in the sural nerve territory.

### Spinal Cord Electrode Implantation and Spinal Cord Stimulation

**FIGURE 1:**
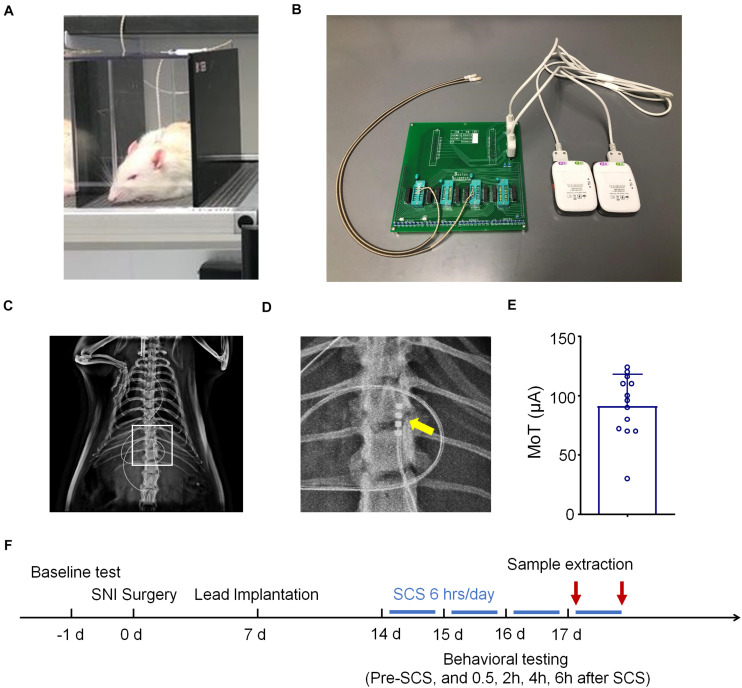
Schematics for spinal cord stimulation (SCS) and experimental design. **(A)** Schematics for SCS and behavioral testing. **(B)** Images of SCS stimulator, which are connected to rats with electrodes. **(C,D)** Radiographs showing electrode implantation within the spinal column of rats. The box in C is enlarged in D with the electrode contacts indicated by an arrow. **(E)** Motor thresholds (MoT) of the rats used in this study, *n* = 13. **(F)** Experimental timeline of SNI surgery, electrode implantation, and SCS.

Electrode implantation was conducted 1 week after nerve injury. A small laminectomy was performed at the T11-T13 vertebral level. The distal end of the electrode was inserted epidurally in the rostral direction. The electrode was then fixed by suture to the muscle, and the proximal end of the electrode was tunneled subdermally, exiting through the skin at the base of the neck (Figure 1B). The proximal end of the electrode was connected to an adapter. The adapter was connected to an external neuro-stimulator and programmer with hardware identical to a commercial device but with firmware and software modified for preclinical use (Boston Scientific) (Figure 1B). To validate the spinal placement of the electrode, radiography was conducted using MultiFocus by a Faxitron system (Faxitron Bioptics LLC, Tucson, AZ, United States) at the Duke University Animal Facility (Figures 1C,D). MT was determined by slowly increasing the current amplitude (4 Hz, 0.25 msec) from zero until an observer saw muscle contraction in the mid-lower trunk or hind limbs under 2% isoflurane (Figure 1E). Rats were assigned either to a “control” group (SNI/SCS Off) or a treatment group (SNI/SCS On). In treatment group animals, SCS was delivered 7–14 days after implantation (2–3 weeks after SNI) as illustrated in Figure 1F. SCS was turned on for 6 h daily, at a frequency of 1,000 Hz, pulse width of 0.1 ms, and using 40 and 80% of the motor threshold (MoT).

### Behavioral Tests for Mechanical and Cold Sensitivity

For testing mechanical sensitivity, rats were put in plastic boxes on an elevated metal mesh floor under stable room temperature and humidity. Rats were habituated to the environment for 2 days before the baseline testing. The hind paws were stimulated with an Electronic von Frey Anesthesiometer (IITC Life Science Inc.) with increasing force (0–50 g), presented perpendicularly to the plantar surface (Shu et al., 2020). The paw withdrawal threshold (PWT) was determined after three trials for each time point. Cold sensitivity was assessed by acetone test. Through the mesh floor two acetone applications (50 μl/application) were gently applied to the bottom of a hindpaw using a pipette (Wang et al., 2020). The duration of time the animal spent lifting and licking the paw over a 90 s period was recorded. Behavioral tests were performed in a blinded manner.

### ELISA Measurement of IL-1β and RvD1

IL-1β ELISA kits were from BioLegend (Catalog number, 432604) and the assay was conducted according to the manufacturer’s protocol. RvD1 ELISA kits from Cayman Chemicals (Catalog number, 500380) were used. For each reaction, 50 μl of serum (collected from tail vein) and 20 μl of CSF (collected from cisterna magna) were collected. Each ELISA was conducted according to the manufacturer’s instruction, and standard curves were included each time. RvD1 ELISA was conducted as previously described (Zhang et al., 2018; Tao et al., 2020) and measured by comparing binding to that of a tracer consisting of RvD1 linked to acetylcholinesterase (AChE) per the manufacturer’s protocol (Cayman Chemicals). The samples and the competition RvD1 tracer were incubated overnight at 4°C. The signal in ELISA plate was developed by Ellman’s Reagent, a substrate of AChE. The optical densities of samples were measured using an ELISA plate reader (Bio-Rad) at a wavelength of 420 nm and RvD1 levels were calculated using the standard curves.

### Drugs and Administration

RvD1 was purchased from Cayman Chemical (CAS Number 872993-05-0). RvD1 was dissolved in PBS containing 10% ethanol. To determine the central action of RvD1, RvD1 (100 and 500 ng) or vehicle (PBS with 10% ethanol) was administered by intrathecal injection. The RvD1 dose was based on our previous study (Zhang et al., 2018; Luo et al., 2019). For intrathecal (i.t.) injection, mice were briefly anesthetized with isoflurane (2%) and a spinal cord puncture was made between the L5 and L6 levels to deliver RvD1 (10 μl) using a 30G needle (Hylden and Wilcox, 1980).

### Statistics

All the data in this study were expressed as mean ± SEM. The sample size for each experiment is indicated in the figure legends and individual data points were indicated when applicable (Figures 3–5). Statistical analyses were conducted with Prism GraphPad 8.3 software. Behavioral data were analyzed using two-tailed student’s *t*-test (two groups), One-Way or Two-Way ANOVA (repeated measures over a time course) followed by *post hoc* Bonferroni test. The criterion for statistical significance was *p* < 0.05.

## Results

### 1 kHz SCS Reduces Mechanical Allodynia in SNI Animals

We implanted one 4-contact electrode per rat in the dorsal epidural space ipsilateral to the side of injury and connected the electrode to an external stimulator (Figures 1A,B). X-Ray autoradiographs confirmed the location of the contacts on the electrode array as ipsilateral at the T12 vertebral level, approximately corresponding with the lumbar enlargement segment of the spinal cord (Figures 1C,D). Figure 1E showed an average MoT of 91.38 ± 7.37 μA (Mean ± SEM, *n* = 13 rats). We delivered SCS 7–14 days after implantation (2–3 weeks after SNI) for a duration of 6 h per day (Figure 1F).

**FIGURE 2:**
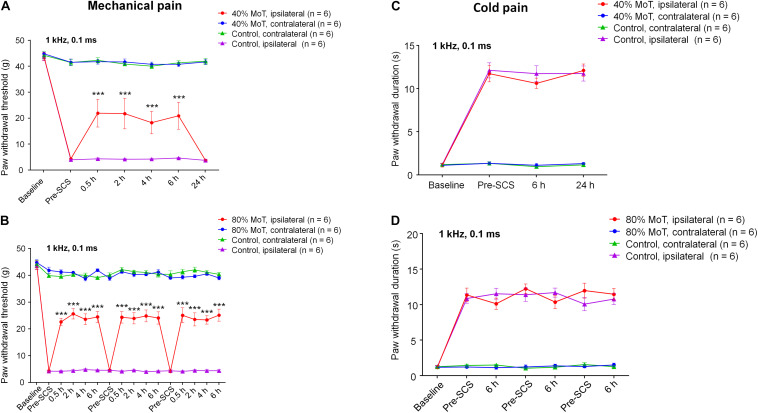
SCS at 1 kHz Hz attenuates nerve injury-induced mechanical allodynia in SNI rats. **(A,C)** Effects of 1 kHz SCS (6 h per day) at 40% MoT on mechanical allodynia measured by paw withdrawal threshold (PWT) using electronic von Frey testing at 0 (Pre-SCS), 0.5, 2, 4, 6, and 24 h **(A)** and cold allodynia in acetone testing at 0 (Pre-SCS), 6, and 24 h **(C)**. **(B,D)** Effects of 1 kHz SCS at 80% MoT on mechanical allodynia in von Frey testing (PWT, **B**) and cold allodynia in acetone testing **(D)**. ***P < 0.001, compared with Pre-SCS baseline. Two-way ANOVA, followed by Bonferroni *post hoc* test. Sample sizes (number of rats per group) are indicated in each panel. SCS was conducted approximately 2 weeks after SNI. Note the SCS has no effects on cold allodynia. Ipsilateral side = injury side. Control animals had SNI surgery and electrode implantation without SCS (SCS-OFF).

Next, we examined the effects of SCS on SNI-induced neuropathic pain (Figures 2A–D). We assessed mechanical sensitivity using an electronic von Frey filament measurement system, showing a mean baseline mechanical threshold of approximately 40 g (Figure 2A). SNI induced robust and sustained mechanical allodynia during the course of this study (2–4 weeks after SNI), as indicated by marked reduction of paw withdrawal threshold (PWT). The PWT reduction was only found on the ipsilateral hind paw but not on the contralateral hindpaw (Figure 2A). SCS (1 kHz, 6 h) at 40% MoT significantly increased PWT; and this increase was observed within 30 min after activation of SCS, was sustained during the entire period of stimulation, but reverted to post-SNI baseline after 24 h (*F*_(18,140)_ = 13.92, *p* < 0.001, Two-way ANOVA, Figure 2A). SNI also caused robust cold hypersensitivity, as indicated by increased withdrawal duration in response to 50 μL of acetone applied to the plantar hindpaw by a pipette (*F*_(9,80)_ = 33.11, *p* < 0.001, Figure 2C).

**FIGURE 3:**
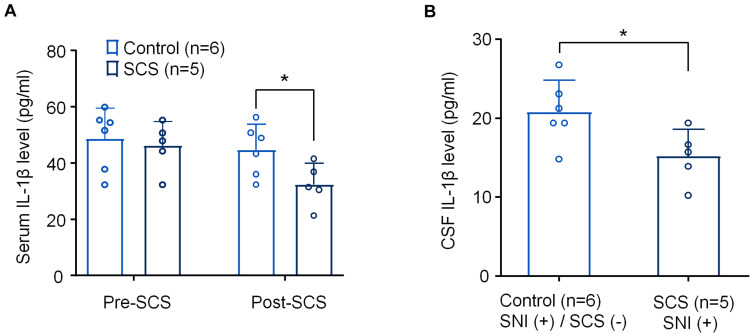
SCS at 1 kHz reduces IL-1β levels in serum and CSF samples from SNI rats. **(A,B)** Effects of 1 k Hz SCS (80% MoT) on IL-1β levels in serum **(A)** and CSF **(B)**. *p < 0.05, One-way ANOVA, followed by Bonferroni *post hoc* test in A. *p < 0.05, two-tailed Student’s *T*-test **(B)**. The sample sizes (number of rats per group) are indicated in each panel. Control = SNI (+)/SCS (−).

**FIGURE 4:**
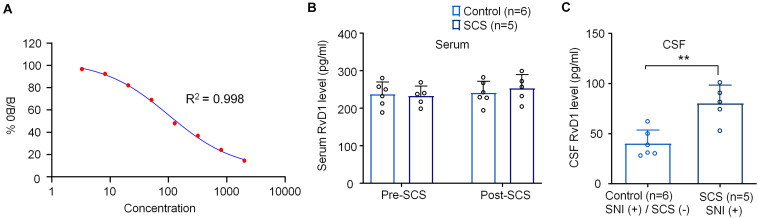
SCS at 1 kHz increases RvD1 levels in CSF samples from SNI rats. **(A)** Standard curve of RvD1 ELISA. *R*^2^ = 0.998. **(B,C)** Effects of 1 kHz SCS (80% MoT) on RvD1 levels in **(B)** serum and **(C)** CSF. **p < 0.01, two-tailed Student’s *T*-test **(C)**. The sample sizes (number of rats per group) are indicated in each panel. Control = SNI (+)/SCS (−).

**FIGURE 5:**
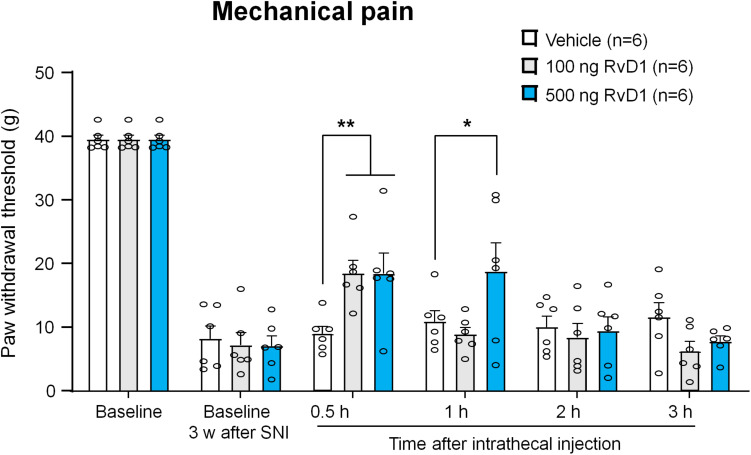
Intrathecal RvD1 reduces SNI-induced mechanical allodynia in rats. RvD1 (100 and 500 ng) was intrathecally injected 3 weeks after SNI. Note that RvD1 produces a dose-dependent inhibition of neuropathic pain. *p < 0.05, **p < 0.01 versus vehicle group. Two-Way ANOVA with Bonferroni’s *post hoc* test, *n* = 6 rats per group.

We also tested 1 kHz SCS at 80% MoT and assessed mechanical and cold sensitivity in the SNI rats. We observed similar increases in PWT during each stimulation (*F*_(45,310)_ = 27.79, *p* < 0.001, Figure 2B). This increase returned to the pre-stimulation baseline at 24 h (Figure 2B). SNI-induced cold pain was not affected by 1 kHz SCS at 80% MoT (*F*_(6,68)_ = 1.193, *p* = 0.3204, Figure 2D). Notably, the acetone test showed that SNI-induced cold pain was not affected by 1 kHz SCS at 40% MoT (*F*_(3,40)_ = 0.3675, *p* = 0.7768, Figure 2C).

### 1 kHz SCS Differentially Regulates IL-1β and RvD1 Levels in Serum and CSF of SNI Animals

We collected serum samples from both the control rats assigned to SNI/SCS-OFF and the treated rats assigned to SNI/SCS-ON both just before applying 1 kHz SCS and 6 h after applying the stimulation (1 kHz, 80% MoT, SCS group). We also collected CSF samples from the SCS group and control group. ELISA analysis revealed a significant reduction of serum IL-1β levels as determined by the Fisher PLSD *post hoc* test (*F*_(1,18)_ = 5.012, *p* < 0.05), Figure 3A. We also saw significant reduction of IL-1β levels in the CSF using unpaired *t*-test (*t* = 2.462, *p* < 0.05, Figure 3B).

We also measured serum and CSF RvD1 levels using ELISA, as we previously demonstrated (Zhang et al., 2018; Tao et al., 2020). Standard curve analysis revealed a reliable measurement of RvD1 with *R*^2^ = 0.998 (Figure 4A). 1 kHz SCS did not change the serum levels of RvD1 (*F*_(1,18)_ = 0.3782, *p* = 0.5463, Figure 4B). Interestingly, the same SCS treatment significantly increased RvD1 in CSF (*t* = 4.194, *p* < 0.01, Figure 4C).

Together, these results suggest (1) SCS differentially regulated IL-1β and RvD1 levels and (2) SCS differentially regulated serum and CSF levels of RvD1.

### Intrathecal RvD1 Reduces SNI-Induced Mechanical Pain in Rats

Because 1 kHz SCS increased RvD1 levels in CSF but not serum samples, central RvD1 may play a role in regulating the SCS-induced pain reduction. To further confirm a role of RvD1 in the SNI-induced neuropathic pain, we treated SNI rats with intrathecal RvD1 (100 and 500 ng) in the late-phase (3 weeks) of neuropathic pain, which is resistant to some anti-inflammatory treatments in the SNI model (Decosterd et al., 2004). We measured PWTs at 0.5, 1, 2, and 3 h after the RvD1 injection. Intrathecal administration of RvD1 significantly increased PWT (*F*_(10,90)_ = 2.831, *p* < 0.01, Two-way ANOVA, Figure 5). RvD1 reduced mechanical allodynia in a dose-dependent manner: 100 ng RvD1 significantly increased PWT at 0.5 h (*p* < 0.01, Bonferroni’s *post hoc* test), while 500 ng RvD1 significantly increased PWT at both 0.5 h and 1 h (*p* < 0.01, *p* < 0.05, Bonferroni’s *post hoc* test) (Figure 5). These results confirmed a role of spinal/central RvD1 in relieving neuropathic pain symptom (mechanical allodynia).

## Discussion

Neuromodulation using spinal cord stimulation (SCS), deep brain stimulation, transcutaneous electrical nerve stimulation, vagus nerve stimulation, and electroacupuncture has been shown to provide pain relief in patients and animals (Han, 2003; Guan et al., 2010; Moreno-Duarte et al., 2014; Pawela et al., 2017; Shamji et al., 2017; Ji et al., 2018; Tao et al., 2020). Recently, neuromodulation was proposed to regulate glial cell function and neuroinflammation in persistent pain conditions (Sato et al., 2014; Ji et al., 2018; Shu et al., 2020; Vallejo et al., 2020). Our results confirm prior studies (Shechter et al., 2013; Song et al., 2014) where the application of a single waveform, in our case an individual 1 kHz SCS waveform, is sufficient to generate significant behavioral analgesia. Furthermore, the reduction of IL-1β levels in serum for the behaviorally effective 1 kHz SCS group also suggests that single tonic SCS waveforms are sufficient for eliciting anti-inflammatory and pro-resolving effects related to microglial activity in the dorsal horn and elsewhere, and such neuroimmune effects play a role in SCS analgesia. As other studies have shown, low frequency (60 Hz) tonic SCS is sufficient to induce morphological changes in microglia and astrocytes that may be associate with a shift from pro-inflammatory to anti-inflammatory cellular polarization (Sato et al., 2014). In light of our results, the 60 Hz results pose the question as to whether a diverse range of more efficient, lower frequency waveforms may represent more optimal paradigms for neuroimmune modulation.

Recent studies also suggested that neuromodulation may increase SPM production. Serhan and collaborators showed that the vagus nerve controls inflammation via the production of SPMs (Serhan et al., 2019). Conversely, vagotomy reduced local production of SPMs and delayed the resolution of inflammation (Mirakaj et al., 2014). Furthermore, the human vagus nerve can produce multiple SPMs including RvE1, RvD5, protectin D1/neuroprotectin D1 (PD1/NPD1), and maresin 1 (MaR1); and intriguingly, electrical stimulation of the vagus nerve not only increased the production of SPMs but also decreased the production of pro-inflammatory prostaglandins and leukotrienes (Serhan et al., 2018, 2019). Neuromodulation via the auricular vagus stimulation through electroacupuncture also resulted in increased production of RvD1 in the dorsal root ganglia after chemotherapy (paclitaxel), which was associated with an alleviation of chemotherapy-induced neuropathic pain by auricular stimulation (Tao et al., 2020).

In this study, we focused on RvD1, partially because it can be readily and reliably measured by ELISA using a commercial kit (Zhang et al., 2018; Tao et al., 2020). RvD1 was highly effective in reducing pain in animal models of inflammatory pain (Xu et al., 2010; Lima-Garcia et al., 2011; Park et al., 2011; Xu and Ji, 2011). Intrathecal RvD1 was shown to potently reduce post-operative pain in rodents. A single RvD1 treatment could prevent the development of post-operative pain after skin-muscle retraction model in rats (Huang et al., 2011). Intrathecal RvD1 post-treatment on post-operative day 9 reduced post-operative pain following thoracotomy that involves nerve injury (Huang et al., 2011; Chi-Fei Wang et al., 2013). Additionally, intrathecal post-treatment of RvD1 (500 ng), at 2 weeks after tibial bone fracture reduced mechanical allodynia and cold allodynia (Zhang et al., 2018). RvD1 has also been shown to reduce neuropathic pain after chemotherapy in mice and rats. Intrathecal RvD1 (100 ng) was effective in attenuating mechanical allodynia 2 weeks after paclitaxel-induced chemotherapy in mice (Luo et al., 2019). Intrathecal administration of aspirin-triggered RvD1 (AT-RvD1, 15 and 150 ng) reduced paclitaxel-evoked hyperactivity of wide-range dynamic (WDR) neurons in the spinal cord; and strikingly, the inhibitory effects of AT-RvD1 on WDR neurons were comparable to that of spinal morphine (Meesawatsom et al., 2020). Additionally, intrathecal administration of RvD1, at an extremely low dose (0.6 pg), decreased hyperalgesia in mice with bone cancer pain (Khasabova et al., 2020). Intrathecal injection of RvD1 (10 or 100 ng) suppressed mechanical allodynia and the up-regulation of TNF-α and IL-1β, while increasing the release of IL-10 and TGF-β1, in a rat model of low-back pain (Liu et al., 2016). Our results showed that intrathecal RvD1 (100 and 500 ng) produced dose-dependent reduction of mechanical allodynia in the rat model of SNI at a late-phase (3 weeks), which is resistant to many anti-inflammatory treatments and nerve blockade (Decosterd et al., 2004; Suter et al., 2009).

It is noteworthy that RvD1 and its precursor DHA, a major component of fish oil, have striking differences in their analgesic actions in late-phase neuropathic pain or post-operative pain. Intrathecal post-treatment (2 weeks after injury) of DHA, at very high doses (500 μg, >1,000 fold of that of RvD1) failed to reduce nerve injury-induced neuropathic pain or bone fracture-induced post-operative pain (Xu et al., 2013; Zhang et al., 2018). However, pre-treatment of DHA via peri-sciatic application or systemic treatment was effective to prevent or delay nerve injury or bone fracture induced neuropathic pain and post-operative pain (Xu et al., 2013; Zhang et al., 2018). Thus, fish oil (DHA) is only effective in pre-treatment for the prevention or delay of the development of chronic pain but is ineffective in the treatment of established pain. In this study, we further demonstrated that high-frequency SCS significantly increased RvD1 secretion in the CSF even following SNI, which was correlated with the analgesic actions of 1 kHz SCS and intrathecal RvD1, suggesting that neurostimulation is an effective adjuvant for resolvin activity in the case of established pain.

In summary, our findings demonstrate that SCS may alleviate neuropathic pain via modulation of neuroinflammation. On the one hand, 1 kHz SCS reduced the IL-1β levels in CSF and serum. IL-1β is sufficient to induce pain hypersensitivity, as well as peripheral sensitization and central sensitization that are essential for the pathogenesis of chronic pain (Milligan et al., 2003; Sommer and Kress, 2004; Binshtok et al., 2008; Kawasaki et al., 2008). Furthermore, 1 kHz SCS also increased the RvD1 levels in the CSF. Given the critical role of RvD1 in the resolution of inflammation and pain, SCS could promote the resolution of neuroinflammation in the CNS. SPMs can be produced by immune cells such as macrophages and interaction of epithelial cells and immune cells from the lipid precursors that are enriched in diet and can also be released from cell membrane lipid layer (Werz et al., 2018). In the CNS, SPMs may also be produced by glial cells such as microglia (Connor et al., 2007; Chen et al., 2018). Notably, spinal cord microglial cells may exhibit different phenotypes such as pro-inflammatory M1-like phenotype and anti-inflammatory M2 phenotypes (Kigerl et al., 2009), as well as pro-resolving phenotype (Chen et al., 2018). Future studies are needed to investigate the outstanding questions: (1) how do microglia produce SPMs and what are the sources (brain, spinal cord, peripheral versus central)? (2) how does SCS modulates microglial phenotypes in neuropathic pain? (3) Do differences in SCS waveform produce different effects on the production and release of SPMs? (4) Are multiple and different SCS dose combinations (pulse rate and pulse width known to be correlated with clinical efficacy (Thomson et al., 2018) enough for neuroimmune modulation and SPM production and release? (5) To what degree do SPMs like RvD1 account for the analgesia produced by SCS? Can RvD1 receptor antagonist block the analgesic action of SCS? (6) In addition to neuropathic pain symptoms, SNI also causes neurological and neuropsychiatric disorders, such as anxiety-like and depressive-like behaviors, and cognitive impairments (Guida et al., 2020). SCS was shown to produce significant improvement in the symptoms of depression and anxiety in patients with failed back surgery (Robb et al., 2017). Does SCS alleviate these co-morbidities of pain via RvD1-mediated neuroinflammation?

## Data Availability Statement

The raw data supporting the conclusions of this article will be made available by the authors, upon request.

## Ethics Statement

The animal study was reviewed and approved by the Institutional Animal Care Use Committee of Duke University.

## Author Contributions

XT and XL did experiments and analyzed the data. R-RJ, TZ, BH, and RE participated in project discussion. R-RJ wrote the manuscript and rest of the authors edited the manuscript. All authors contributed to the article and approved the submitted version.

## Conflict of Interest

R-RJ is a consultant of Boston Scientific and he also received a grant from the company. TZ, BH, and RE are employees of Boston Scientific. This is a mechanistic study in animals and does not involve new product from the company. The remaining authors declare that the research was conducted in the absence of any commercial or financial relationships that could be construed as a potential conflict of interest.
